# The Multilevel Effects of Principals’ Servant Leadership on Kindergarten Teachers’ Job Crafting: The Mediating Role of Organizational Identification

**DOI:** 10.3390/bs16030329

**Published:** 2026-02-27

**Authors:** Xiaoqing Lin, Runkai Jiao, Feifei Li

**Affiliations:** 1 School of Education and Psychology, Minnan Normal University, Zhangzhou 363000, China; linxq799@nenu.edu.cn; 2 School of Psychology, Northeast Normal University, Changchun 130024, China; jiaork@nenu.edu.cn; 3 College of Education, Wenzhou University, Wenzhou 325035, China

**Keywords:** servant leadership, approach job crafting, avoidance job crafting, organizational identification, multilevel analysis

## Abstract

Job crafting has become an essential strategy for kindergarten teachers to cope with increasing job demands and sustain professional engagement. Drawing on the proactive motivation model, this study examines whether and how principals’ servant leadership exerts cross-level effects on teachers’ approach and avoidance job crafting. Data were collected from 1724 teachers nested within 150 kindergartens, and hypotheses were tested using multilevel modeling. The results indicated that principals’ servant leadership had significant cross-level effects on teachers’ approach and avoidance job crafting, positively predicting approach job crafting and negatively predicting avoidance job crafting. In addition, organizational identification functioned as a cross-level mediator in this relationship, through which servant leadership further enhanced approach job crafting and reduced avoidance job crafting. These findings extend the literature by revealing the motivational pathway linking servant leadership to distinct forms of job crafting and highlight the importance of cultivating a servant leadership climate to foster proactive behaviors among kindergarten teachers.

## 1. Introduction

Teachers are essential to educational quality, with kindergarten teachers playing a key role in promoting early childhood education (Xia et al., 2024). Their work extends beyond instruction to include childcare and a range of non-instructional duties (Hargreaves, 2000). Yet kindergarten teachers often face substantial job pressures, such as relatively low pay and heavy workloads. These pressures may undermine job stability, constrain professional development (Y. Wang et al., 2024), and heighten turnover intentions (Ma, 2022; Ren et al., 2024). In this context, job crafting is viewed as an important self-regulatory strategy for managing occupational challenges. It involves proactively modifying tasks, relationships, and cognitive perceptions to align work with one’s goals and competencies (Wrzesniewski & Dutton, 2001). Job crafting has been associated with better teacher well-being and lower work-related strain (Chen et al., 2021; Y. Wang et al., 2024; Zheng et al., 2023). It is commonly conceptualized as comprising two distinct forms: approach job crafting (AJC) and avoidance job crafting (AvJC).

AJC involves proactive efforts aimed at gaining desirable outcomes (e.g., greater fulfillment, well-being, and performance), whereas AvJC involves efforts aimed at reducing or avoiding undesirable aspects of work and has been associated with less favorable experiences or outcomes (Bruning & Campion, 2018; F. Zhang & Parker, 2019). Given their conceptual distinctiveness and potentially different antecedents, scholars have called for examining AJC and AvJC separately (Tian et al., 2020). Empirical evidence has begun to delineate their distinct correlates and consequences (Zhou et al., 2025). Extending this line of inquiry to early childhood education, a critical gap remains in clarifying factors that promote AJC and reduce AvJC among kindergarten teachers.

Proactive behaviors such as job crafting are influenced by interpersonal interactions, particularly with leaders (Xue & Woo, 2022). Leadership can either encourage or hinder employees’ initiative (Hamid, 2025; H. Wang et al., 2020; F. Zhang & Parker, 2019). This is especially salient in kindergartens, where daily work is highly relational and principals play a central role in influencing teachers’ work experiences and behavioral responses. Servant leadership, which emphasizes supporting, caring for, and developing followers (Y. Wang et al., 2023), may be particularly relevant in this context. By showing respect, granting autonomy, and fostering a sense of responsibility, servant leaders can cultivate a psychologically safe and empowering climate that encourages teachers to proactively adjust their roles and work practices. Prior research indicates that servant leadership is associated with better employee outcomes (Al-Asadi et al., 2019), helps align employees’ efforts with organizational goals (Eva et al., 2019; Liden et al., 2008), and may facilitate job crafting (Ni et al., 2022).

Leadership can exert influence at multiple levels, including a shared leadership climate at the team level and employees’ individual perceptions of leadership (F. Zhang & Liao, 2016). Multilevel theory suggests that single-level designs cannot adequately examine the interplay between contextual factors and individual characteristics (Gong et al., 2013; P. Ye et al., 2022). Accordingly, scholars have called for multilevel methods in job crafting research to clarify how contextual factors are associated with employees’ job crafting (Harju et al., 2018; Tims et al., 2022). Existing multilevel research has largely concentrated on team-level servant leadership in relation to AJC, whereas AvJC has received relatively less attention. In addition, the mechanisms underlying team-level servant leadership’s relationships with both AJC and AvJC remain unclear. Leadership effects are also context-dependent, and organizational settings may influence the magnitude and generalizability of these relationships (Oc, 2018; Liden & Antonakis, 2009). Therefore, the present study focuses on the kindergarten context to examine the cross-level relationships between team-level servant leadership and teachers’ AJC and AvJC.

Drawing on the proactive motivation model, this study examines how team-level servant leadership is associated with kindergarten teachers’ AJC and AvJC. The model proposes that contextual factors influence proactive motivational states (“can do,” “reason to,” and “energized to”), which in turn drive proactive behaviors such as job crafting (Fong et al., 2022; Parker et al., 2010). Among these states, the “reason to” motivational state is particularly important in proactive goal pursuit. It reflects whether individuals view proactive behavior as meaningful or normatively expected, which can sustain their willingness to maintain proactive efforts (Parker et al., 2010; Xu et al., 2025). In kindergarten settings, teachers often need to adjust their work methods and practices in response to children’s developmental needs, classroom management demands, and organizational goals (Hargreaves, 2000). Whether teachers are willing to sustain such proactive adjustments may depend on whether they develop a sense of belongingness and responsibility toward the kindergarten, namely organizational identification. Organizational identification can be conceptualized as a “reason to” motivational state (Parker et al., 2010; Xu et al., 2025). Thus, this study treats organizational identification as a mediating mechanism between team-level servant leadership and teachers’ AJC and AvJC.

The present study contributes to the servant leadership and job crafting literature in two ways. First, adopting a multilevel perspective, we examine the cross-level associations between team-level servant leadership and teachers’ AJC and AvJC. This extends previous research that has primarily focused on AJC and highlights servant leadership’s differentiated links with the two forms of job crafting. Second, grounded in the proactive motivation model, we test organizational identification as a “reason to” motivational mechanism linking servant leadership with teachers’ AJC and AvJC.

In sum, this study examines the relationships between team-level servant leadership and kindergarten teachers’ AJC and AvJC and investigates the mediating role of organizational identification. Specifically, this study addresses the following research questions: (1) How is team-level servant leadership associated with kindergarten teachers’ AJC and AvJC? (2) Does organizational identification mediate the relationships between team-level servant leadership and kindergarten teachers’ AJC and AvJC?

The remainder of this article is organized as follows. Section 2 presents the theoretical background and hypothesis development. Section 3 describes the participants, procedure, measures, and analytic strategy. Section 4 reports the results. Section 5 discusses the findings. Section 6 concludes the paper.

## 2. Theoretical Background and Research Hypothesis

### 2.1. Approach Job Crafting and Avoidance Job Crafting

Job crafting refers to employees’ self-initiated changes to their work design to better align job characteristics with their skills, abilities, and preferences (Tims et al., 2022). Prior research has mainly conceptualized job crafting from two perspectives: the role perspective and the resource perspective. The role perspective distinguishes three forms of crafting (Wrzesniewski & Dutton, 2001): task crafting (changing work activities), relational crafting (adjusting workplace interactions), and cognitive crafting (reconceptualizing the meaning of the job). The resource perspective distinguishes four strategies: increasing structural resources (e.g., greater autonomy or skill development), increasing social resources (e.g., support from colleagues), increasing challenging job demands (e.g., taking on additional tasks or responsibilities), and decreasing hindering job demands (e.g., reducing cognitive or emotional workload) (Tims et al., 2012).

Given that these two perspectives overlap conceptually, it is necessary to integrate them. Accordingly, job crafting is often differentiated into two higher-order forms: AJC and AvJC (Bruning & Campion, 2018; F. Zhang & Parker, 2019). AJC involves proactive changes oriented toward positive outcomes (e.g., seeking resources, taking on challenges, and pursuing growth). In contrast, AvJC involves changes aimed at reducing or avoiding undesirable aspects of work (e.g., decreasing hindering job demands) (F. Zhang & Parker, 2019; Lopper et al., 2023). Parker et al. (2019) proposed the idea of “wise proactivity,” suggesting that wise proactive behavior should take into account the needs of the individual, the task, and the broader context. AJC is more likely to meet these three needs and is more consistent with constructive, growth-oriented proactive behavior. In contrast, although AvJC may reduce strain in the short term, in some situations it may also come with potential costs for task effectiveness or collaboration (Parker et al., 2019; Fong et al., 2021). Given that AJC and AvJC differ in motivational focus and outcomes, examining the two forms can provide a more complete understanding of kindergarten teachers’ job crafting (Lin et al., 2023, 2025).

### 2.2. Servant Leadership, Approach Job Crafting, and Avoidance Job Crafting

Servant leadership is a follower-centered leadership style that prioritizes employees’ needs, growth, and intrinsic motivation and builds trust by helping employees experience greater meaning in their work (Liden et al., 2008; van Dierendonck, 2011). It comprises seven dimensions: conceptual skills, empowerment, helping subordinates grow and succeed, ethical behavior, putting subordinates first, creating value for the community, and building relationships (Liden et al., 2008). Unlike leadership styles that rely primarily on formal authority, servant leadership supports followers through empowerment, ethical conduct, and an emphasis on community-oriented values (Eva et al., 2019; Sendjaya et al., 2008). As an important contextual resource, servant leadership can provide social and psychological resources such as autonomy, support, and development opportunities, which may foster proactivity and positive work attitudes (Adams et al., 2025; Eva et al., 2019).

According to the proactive motivation model, leadership is a key contextual factor that can shape proactive behaviors such as job crafting (Bavik et al., 2017; Harju et al., 2018). In team settings, teachers who work with the same principal are likely to develop shared perceptions of the principal’s leadership, and such team-level perceptions can have cross-level associations with teachers’ attitudes and behaviors (Kozlowski & Klein, 2000). Prior cross-level studies have shown that team-level servant leadership is positively related to AJC (Bavik et al., 2017; Harju et al., 2018). Moreover, servant leadership has been linked to fewer negative or withdrawal-related behaviors (Anwaar & Jingwei, 2022). In particular, servant leadership is negatively related to demand reduction behaviors (Tuan, 2022), which conceptually align with AvJC because they reflect efforts to minimize or avoid undesirable job demands (Bruning & Campion, 2018; F. Zhang & Parker, 2019). By fostering trust, psychological safety, and resource support, servant leadership may be associated with a reduced perceived need for defensive, avoidance-oriented adjustments. Based on these arguments, we propose the following hypotheses:

**H1a.**  *Team-level servant leadership is positively associated with kindergarten teachers’ AJC*.

**H1b.**  *Team-level servant leadership is negatively associated with kindergarten teachers’ AvJC*.

### 2.3. Mediating Role of Organizational Identification

Organizational identification refers to the extent to which individuals define themselves in terms of their organization and develop a sense of belonging to the organization (Mael & Ashforth, 1992). It provides identity-based value that can shape individuals’ affect, cognition, and behavior (Tajfel & Turner, 1979). It emerges as individuals incorporate organizational beliefs and values into their self-concept and is strengthened through ongoing organizational interactions (Albert & Whetten, 1985; McAllister & Bigley, 2002). According to the proactive motivation model, the “reason to” motivational state concerns whether proactive action is experienced as meaningful and normatively appropriate (Parker et al., 2010). By fostering a sense of responsibility and purpose toward the organization, organizational identification may facilitate the internalization of organizational goals as reasons worth sustained effort. Organizational identification can be viewed as an expression of the “reason to” motivational state (Xu et al., 2025).

The proactive motivation model suggests that contextual factors such as leadership can affect individuals’ motivational states, which may in turn be associated with subsequent proactive behavior. Previous research has shown that servant leadership is positively related to employees’ organizational identification (Akbari et al., 2014; Omanwar & Agrawal, 2022; B. Ye et al., 2020). Servant leadership emphasizes care, respect, and empowerment. By supporting employees’ growth and development, servant leaders communicate that employees are valued and supported. Such experiences may foster positive evaluations of the organization and a stronger sense of belonging, thereby enhancing organizational identification. In the kindergarten context, principals often embody the organization’s culture and values. Servant leadership may convey organizational values and expected behaviors (Zhu et al., 2015). This may further strengthen teachers’ organizational identification. When organizational identification is stronger, individuals are more likely to engage in proactive behaviors that align with organizational goals and role expectations (Gao & Zhao, 2014; Liu et al., 2019). Prior research also suggests that organizational identification is positively related to AJC (Sameer & Priyadarshi, 2021). Accordingly, when teachers strongly identify with their kindergarten and internalize its values, they may be more likely to engage in AJC. In contrast, AvJC involves adjustments intended to reduce or avoid unfavorable aspects of work. Organizational identification is often associated with lower withdrawal tendencies (S. Zhang & Liu, 2016). Thus, teachers with higher organizational identification may be less likely to engage in AvJC. Based on these arguments, we propose the following hypotheses:

**H2a.**  *Organizational identification mediates the positive association between team-level servant leadership and kindergarten teachers’ AJC*.

**H2b.**  *Organizational identification mediates the negative association between team-level servant leadership and kindergarten teachers’ AvJC*.

**Figure 1:**
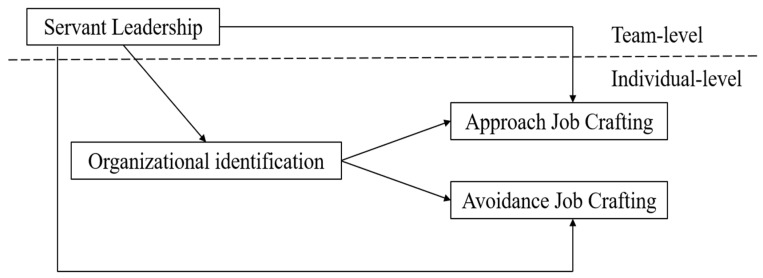
The theoretical framework linking principals’ servant leadership to kindergarten teachers’ approach and avoidance job crafting.

The theoretical hypothesis model of this study is shown in Figure 1.

## 3. Materials and Methods

### 3.1. Participants and Procedure

Data for this study were collected through the National Kindergarten Principal Training Center, an institution responsible for the professional development of kindergarten principals across China. As a regular trainer at the center, the second author randomly invited principals from diverse geographical regions across China, including eastern (e.g., Zhejiang), central (e.g., Hubei, Shanxi), and western (e.g., Sichuan, Shaanxi, Chongqing) areas, to distribute the online survey link to teachers in their kindergartens. On the first page of the online questionnaire, detailed instructions and an informed consent form were provided. Participants were informed that their responses would remain anonymous, the study was conducted for academic purposes, they had the right to withdraw at any time without penalty, and all data would be handled with strict confidentiality. Only teachers who consented proceeded to complete the questionnaire. This study was approved by the Ethics Committee of the second author’s university.

A total of 2012 responses were collected from teachers across 170 kindergartens. After data screening, responses that showed patterned answering or failed the attention-check item were removed, resulting in 1816 valid questionnaires. To ensure the reliability of multilevel analyses, only kindergartens with at least five valid responses were included in the final sample (Mathieu et al., 2012), yielding 1724 valid questionnaires from 150 kindergartens. The valid data of each kindergarten ranged from 5 to 20 teachers, with an average of 11.5 teachers per kindergarten. Among the valid sample, the participating teachers had an average age of 32.83 years (SD = 7.89, range 20–57 years) and an average teaching experience of 9.45 years (SD = 8.25, range 1–37 years). In terms of educational attainment, 646 teachers (37.50%) had an associate degree or lower, and 1078 (62.50%) held a bachelor’s degree or higher. Regarding kindergarten location, 1435 teachers (83.20%) worked in urban areas and 289 (16.80%) in township areas.

### 3.2. Measures

#### 3.2.1. Approach–Avoidance Job Crafting

Kindergarten teachers’ job crafting was assessed using the 29-item scale developed by Lin (2025). This scale comprises two subscales: approach job crafting (AJC) and avoidance job crafting (AvJC), each with three dimensions (i.e., task, relational, and cognitive crafting). An example item for AJC is “I actively look for various teaching materials to enrich my daily teaching,” and an example item for AvJC is “I try to avoid changing my daily teaching activities and methods.” Responses were rated on a five-point scale (1 = never, 5 = always), with higher scores indicating greater frequency of job crafting behaviors. In the present study, Confirmatory factor analysis showed acceptable model fits for both subscales: AJC, χ^2^/df = 10.829, SRMR = 0.076, RMSEA = 0.054, CFI = 0.895, TLI = 0.875; AvJC, χ^2^/df = 6.957, SRMR = 0.059, RMSEA = 0.040, CFI = 0.954, TLI = 0.942. Cronbach’s α coefficients were as follows: for AJC, α = 0.91 for the total scale, and 0.85 (task crafting), 0.83 (relational crafting), and 0.87 (cognitive crafting) for its dimensions; for AvJC, α = 0.90 for the total scale, and 0.82 (task crafting), 0.89 (relational crafting), and 0.87 (cognitive crafting) for its dimensions.

#### 3.2.2. Servant Leadership

Servant leadership was measured using the 7-item scale revised by Gao and Zhao (2014). Each item of this scale assesses one of the following seven dimensions: empowerment, putting followers first, emotional healing, helping followers grow and succeed, ethical behavior, conceptual skills, and creating value for the community. To ensure contextual relevance, the term “leader” in items was replaced with “principal” to align with the kindergarten setting. An example item is: “My principal gives me autonomy to deal with challenges in the best way.” The items were rated on a 7-point Likert scale (1 = strongly disagree, 7 = strongly agree). In the current study, Confirmatory factor analysis showed a good model fit: χ^2^/df = 14.683, SRMR = 0.039, RMSEA = 0.089, CFI = 0.937, TLI = 0.906. Cronbach’s α coefficient for the total scale was 0.88.

#### 3.2.3. Organizational Identification

Organizational identification was measured using the unidimensional scale developed by Mael and Ashforth (1992), which consists of six items. An example item is: “When someone praises my kindergarten, I feel personally recognized.” To ensure contextual relevance, the term “organization/company” in the original scale was replaced with “kindergarten.” Participants rated each item on a 5-point scale (1 = strongly disagree, 5 = strongly agree), with higher scores indicating stronger organizational identification. In the current study, Confirmatory factor analysis showed a good model fit: χ^2^/df = 9.563, SRMR = 0.026, RMSEA = 0.070, CFI = 0.955, TLI = 0.925. Cronbach’s α coefficient for the total scale was 0.88.

#### 3.2.4. Control Variables

Prior research suggests that job crafting may be associated with various demographic factors, such as age, education, and work experience (Lin et al., 2025; Rudolph et al., 2017). Given these findings, the present study controlled for these variables in all analyses.

### 3.3. Statistical Analysis

Given that the 1724 teachers in this study were nested within 150 kindergartens, data were analyzed using Mplus 8.3 and R Studio 4.0.4 to account for the hierarchical structure of the data. First, a common method bias test was conducted. Second, confirmatory factor analyses (CFA) were conducted to examine the discriminant validity of the study variables. Factor loadings, composite reliability (CR), and average variance extracted (AVE) were reported. Third, correlation analyses were performed to examine the relationships among the variables. Fourth, data aggregation tests were conducted for servant leadership. Because servant leadership was assessed based on teachers’ perceptions, it was necessary to evaluate within-group agreement among teachers in the same kindergarten before aggregating individual-level responses to the team level. The within-group agreement index Rwg was used for this purpose. An Rwg value exceeding 0.70 indicates sufficient consistency to justify aggregation (James et al., 1993). Fifth, three unconditional models were estimated with organizational identification, AJC, and AvJC as the dependent variables to assess between-group variance. Using the intraclass cor-relation coefficient formula ICC(1) = intraclass variance (σ^2^)/[interclass variance (τ^2^) + intraclass variance (σ^2^)], an ICC(1) value greater than 0.05 indicates significant between-group variability and supports the use of multilevel modeling (Cohen, 1988). ICC(2), representing the reliability of group means, was above 0.50, suggesting that the aggregated group-level construct demonstrated adequate reliability (Bliese, 2000). Finally, multilevel regression analyses were conducted to test the hypotheses, controlling for age, work experience, and education. Individual-level predictors were group-mean centered, and team-level predictors were grand-mean centered.

## 4. Results

### 4.1. Common Method Variance Test

To minimize the potential influence of common method variance, we implemented procedural remedies during data collection, including emphasizing the confidentiality and anonymity of the survey to reduce social desirability and evaluation apprehension. For the statistical assessment, we first conducted Harman’s single-factor test. The results showed that the single-factor model fit the data poorly: χ^2^/df = 27.612, SRMR = 0.150, RMSEA = 0.124, CFI = 0.348, and TLI = 0.315. Given the limited power of this test (Tang & Wen, 2020), we conducted a more rigorous analysis by adding an unmeasured latent method construct (ULMC) (Podsakoff et al., 2003). The ULMC model showed the following fit indices: χ^2^/df = 4.159, SRMR = 0.060, RMSEA = 0.043, CFI = 0.921, and TLI = 0.915, whereas the four-factor measurement model showed: χ^2^/df = 4.534, SRMR = 0.050, RMSEA = 0.045, CFI = 0.915, and TLI = 0.909. The comparison indicated that the changes in RMSEA and SRMR were both below 0.05, and the changes in CFI and TLI were both below 0.10, suggesting that adding the method factor did not substantially improve model fit. Therefore, these results indicate that common method bias did not significantly affect the subsequent analyses in this study.

### 4.2. Confirmatory Factor Analysis

As shown in Table 1, the four-factor model (SL, OI, AJC, AvJC) showed a better fit to the data than the alternative models, including the three-factor solution (SL, OI, AJC + AvJC), the two-factor solution (SL + OI, AJC + AvJC), and the single-factor solution (SL + OI + AJC + AvJC). The findings indicate good discriminant validity among the four constructs, supporting the subsequent analyses.

Table 2 summarizes the reliability and convergent validity results for each construct. Standardized factor loadings ranged from 0.53 to 0.90. CR values ranged from 0.89 to 0.95, exceeding the commonly recommended threshold of 0.70 (George & Mallery, 2003). AVE values ranged from 0.54 to 0.64, exceeding the 0.50 criterion (Hair et al., 1998). Overall, these results suggest adequate reliability and convergent validity for the constructs.

### 4.3. Correlation Analysis

Servant leadership was positively correlated with organizational identification and AJC, and negatively correlated with AvJC (see Table 3). Organizational identification was positively correlated with AJC and negatively correlated with AvJC. Age was positively correlated with servant leadership, work experience was positively correlated with organizational identification, and education level was positively correlated with AvJC.

### 4.4. Data Aggregation Tests

Consistent with prior research (e.g., Harju et al., 2018; P. Ye et al., 2022), principal servant leadership was conceptualized as a shared team climate within each kindergarten; therefore, teachers’ ratings were aggregated to the team level. The results indicated high within-team agreement (mean Rwg = 0.89, range = 0.77–0.99), exceeding the 0.70 threshold. In addition, servant leadership showed meaningful between-team variability (ICC(1) = 0.109), exceeding the 0.05 threshold, and the reliability of the team means was acceptable (ICC(2) = 0.604), exceeding the 0.50 threshold. These indices provide empirical support for aggregating servant leadership as a team-level variable and for conducting multilevel analyses.

### 4.5. Hypotheses Testing

Before conducting the multilevel analyses, we specified three unconditional models with organizational identification, AJC, and AvJC as the dependent variables, respectively, to examine the extent of between-group variance in these individual-level variables. The results showed between-group variance for all three outcomes, with ICC(1) values of 0.102 for organizational identification (σ^2^ = 0.407; τ^2^ = 0.046), 0.156 for AJC (σ^2^ = 0.228; τ^2^ = 0.042), and 0.125 for AvJC (σ^2^ = 0.478; τ^2^ = 0.068). As Cohen (1988) suggested, the ICC(1) values for organizational identification, AJC, and AvJC were all above 0.05, indicating sufficient between-group variance to justify the use of multilevel analysis.

The multilevel results are reported in Table 4. In the models without the mediator, team-level servant leadership was positively associated with teachers’ AJC (Model 1: B = 0.32, *p* < 0.001) and negatively associated with AvJC (Model 2: B = −0.28, *p* < 0.001), supporting H1a and H1b. Servant leadership was also positively associated with teachers’ organizational identification (Model 3: B = 0.43, *p* < 0.001). When organizational identification was included in the model, both servant leadership (Model 4: B = 0.22, *p* < 0.001) and organizational identification (B = 0.23, *p* < 0.001) were positively associated with AJC. For AvJC, both servant leadership (Model 5: B = −0.22, *p* < 0.001) and organizational identification (B = −0.14, *p* < 0.001) were negatively associated with AvJC. These results suggest an indirect pathway from servant leadership to both forms of job crafting through organizational identification. The explained variance increased after adding organizational identification (AJC: R^2^ = 0.15 in Model 1 to 0.23 in Model 4; AvJC: R^2^ = 0.13 in Model 2 to 0.14 in Model 5). The slopes for organizational identification varied across kindergartens (random-slope variance = 0.013, SD = 0.113 for AJC; 0.030, SD = 0.175 for AvJC), suggesting that the strength of the association between organizational identification and job crafting varies across kindergartens.

To further test the mediation hypotheses, we estimated the indirect effects using the bruceR package with quasi-Bayesian MCMC 95% credible intervals. For AJC, the indirect effect of team-level servant leadership on AJC through organizational identification was significant (B = 0.10, SE = 0.01, *p* < 0.001; MCMC 95% CI = [0.08, 0.12]). The direct effect remained significant (B = 0.23, SE = 0.03, *p* < 0.001; MCMC 95% CI = [0.16, 0.29]), indicating partial mediation. The proportion mediated by organizational identification was 30.30%, supporting H2a.

For AvJC, the indirect effect through organizational identification was also significant (B = −0.06, SE = 0.01, *p* < 0.001; MCMC 95% CI = [−0.08, −0.03]). The direct effect remained significant (B = −0.22, SE = 0.05, *p* < 0.001; MCMC 95% CI = [−0.32, −0.12]), indicating partial mediation. The proportion mediated by organizational identification was 21.40%, supporting H2b.

## 5. Discussion

Building on the proactive motivation model, we examined the associations between team-level servant leadership and kindergarten teachers’ AJC and AvJC. Results showed positive associations with AJC and negative associations with AvJC, and these associations may be partially mediated by teachers’ organizational identification.

The results of this study showed that team-level servant leadership was positively associated with kindergarten teachers’ AJC and negatively associated with AvJC. The finding for AJC is consistent with prior research (Bavik et al., 2017; Harju et al., 2018), suggesting that in the highly interactive work context of early childhood education, servant leadership may be accompanied by a supportive, growth-oriented team climate that encourages teachers to make constructive adjustments by seeking resources and embracing challenges. In contrast, AvJC refers to adjustments aimed at reducing or avoiding unpleasant aspects of work (F. Zhang & Parker, 2019). In a more supportive and growth-focused leadership climate, teachers may feel less threatened and uncertain and therefore be less likely to adopt avoidance-oriented adjustments. It should be emphasized that a reduction in AvJC does not necessarily imply more positive outcomes, as prior research suggests that its effects may vary depending on its specific form and the work design context (Lopper et al., 2025).

This study also found that team-level servant leadership was indirectly related to both forms of teachers’ job crafting through organizational identification. Specifically, servant leadership was positively associated with organizational identification, consistent with prior research (Akbari et al., 2014; Omanwar & Agrawal, 2022; B. Ye et al., 2020). This suggests that the caring, respect, and support for teachers’ development reflected in servant leadership may be linked to teachers’ stronger sense of belonging and value alignment with their kindergarten. When teachers identify more strongly with their kindergarten, they are more likely to view efforts such as seeking resources and embracing challenges as meaningful and consistent with organizational expectations, and thus engage more in AJC and less in AvJC. This is also in line with evidence that organizational identification is often related to more positive attitudes and more constructive behavioral tendencies (Gao & Zhao, 2014; Liu et al., 2019). It should be noted that in the present study, organizational identification was negatively associated with AvJC, which differs from the findings of Sameer and Priyadarshi (2021). The study showed that organizational identification was positively associated with decreasing hindering job demands, which can be considered a form of AvJC (Bruning & Campion, 2018; F. Zhang & Parker, 2019). This difference may reflect contextual differences. In settings with higher job security or stronger organizational constraints, employees may be more likely to manage demands by reducing hindering job demands, which could lead to a pattern different from that observed in the present study.

## 6. Conclusions

This study found that team-level servant leadership was positively associated with kindergarten teachers’ AJC and negatively associated with their AvJC, and was indirectly associated with both forms of job crafting through organizational identification. This study makes two theoretical contributions. First, it reveals differentiated associations between team-level servant leadership and two forms of job crafting, underscoring the importance of distinguishing job crafting orientations in leadership research. Second, by examining the indirect pathway of servant leadership–organizational identification–job crafting, this study extends the applicability of the proactive motivation model to the kindergarten context. Practically, the findings suggest that kindergartens may benefit from strengthening principals’ servant leadership practices (e.g., empowering teachers, leading by example, and supporting teachers’ professional growth) and enhancing teachers’ organizational identification (e.g., through participation opportunities, recognition, and adequate resources), which together are associated with higher AJC and lower AvJC.

This study has several limitations. First, because the data were cross-sectional and collected from a single source, the findings reflect associations rather than allowing strong causal inference or definitive conclusions about mediation, and common method bias cannot be fully ruled out despite procedural remedies and statistical checks. Future research should adopt longitudinal, experimental, or quasi-experimental designs and incorporate multi-source data to more rigorously test the proposed mechanisms. Second, servant leadership was assessed only via teachers’ reports; future studies could triangulate ratings from principals, peers, or third parties to strengthen measurement robustness. Third, the sample was primarily recruited through training networks, which may limit representativeness; future work should broaden recruitment channels and include kindergartens of different types and teachers at different career stages. Finally, the study was conducted in China, and the generalizability of the findings should be interpreted with caution. Replications across cultural and institutional contexts, along with tests of theoretically relevant boundary conditions (e.g., promotion focus, job autonomy, resource availability, and workload), would help clarify when and for whom these relationships are more likely to emerge. Future research could also refine the measures and conduct cross-sample validation to further enhance the robustness of the conclusions.

## Figures and Tables

**Table 1:** Model fit indicators.

Models	χ^2^	df	χ^2^/df	CFI	TLI	RMSEA	SRMR
4-factor model (SL, OI, AJC, AvJC)	3658.947	807	4.534	0.915	0.909	0.045	0.050
3-factor model (SL, OI, AJC + AvJC)	6662.075	810	8.224	0.825	0.814	0.065	0.070
2-factor model (SL + OI, AJC + AvJC)	7536.139	812	9.280	0.799	0.787	0.069	0.103
1-factor model (SL + OI + AJC + AvJC)	22,614.868	819	27.612	0.348	0.315	0.124	0.150

Note: χ^2^ = chi-square; df = degrees of freedom; CFI = comparative fit index; TLI = Tucker–Lewis index; RMSEA = root-mean-square error of approximation; SRMR = standardized root-mean square residual. SL, servant leadership; OI, organizational identification; AJC, Approach Job Crafting; AvJC, Avoidance Job Crafting.

**Table 2:** Scales’ reliability and validity.

Variable	Factor Loading	CR	AVE
servant leadership	0.53~0.84	0.89	0.56
organizational identification	0.71~0.88	0.91	0.64
approach job crafting	0.66~0.85	0.94	0.54
avoidance job crafting	0.57~0.90	0.95	0.61

**Table 3:** Descriptive statistics and correlations among study variables.

	M	SD	1	2	3	4	5	6	7
1 Age	32.83	7.89	1						
2 Work experience	9.45	8.25	0.81 ***	1					
3 Education	0.62	0.48	0.001	0.11 ***	1				
4 SL	5.35	1.07	0.05 *	0.01	−0.04	1			
5 OI	4.34	0.67	0.17 ***	0.15 ***	0.04	0.52 ***	1		
6 AJC	4.12	0.51	0.03	0.03	0.03	0.39 ***	0.36 ***	1	
7 AVJC	2.42	0.73	−0.03	0.04	0.10 ***	−0.16 ***	−0.16 ***	−0.20 ***	1

Note: *N* = 1724. * *p* < 0.05, *** *p <* 0.001. SL, servant leadership; OI, organizational identification; AJC, approach job crafting; AVJC, avoidance job crafting.

**Table 4:** Results of the multilevel analysis.

	AJC		AVJC		OI		AJC		AVJC	
	Model 1		Model 2		Model 3		Model 4		Model 5	
	B	SE	B	SE	B	SE	B	SE	B	SE
Level 1 (*N* = 1724)										
Intercept	4.17 ***	0.02	2.42 ***	0.02	4.34 ***	0.02	4.12 ***	0.02	2.42 ***	0.02
Age	−0.001	0.002	−0.01 *	0.004	0.01 **	0.003	−0.004	0.002	−0.01 *	0.004
Work experience	0.003	0.002	0.01 *	0.004	0.003	0.003	0.002	0.003	0.01 *	0.004
Education	0.04	0.03	0.11 **	0.04	0.06 *	0.03	0.03	0.02	0.12 **	0.04
OI							0.23 ***	0.02	−0.14 ***	0.03
Level 2 (*N* = 150)										
SL	0.32 ***	0.03	−0.28 ***	0.05	0.43 ***	0.03	0.22 ***	0.03	−0.22 ***	0.05
*R* ^2^	0.15		0.13		0.13		0.23		0.14	

Note: * *p* < 0.05, ** *p* < 0.01, *** *p* < 0.001. SL, servant leadership; OI, organizational identification; AJC, approach job crafting; AVJC, avoidance job crafting.

## Data Availability

The data that supports the findings of this study are contained within the article and available from the first author upon reasonable request.
